# Two Poplar-Associated Bacterial Isolates Induce Additive Favorable Responses in a Constructed Plant-Microbiome System

**DOI:** 10.3389/fpls.2016.00497

**Published:** 2016-04-26

**Authors:** Collin M. Timm, Dale A. Pelletier, Sara S. Jawdy, Lee E. Gunter, Jeremiah A. Henning, Nancy Engle, Jayde Aufrecht, Emily Gee, Intawat Nookaew, Zamin Yang, Tse-Yuan Lu, Timothy J. Tschaplinski, Mitchel J. Doktycz, Gerald A. Tuskan, David J. Weston

**Affiliations:** ^1^Biosciences Division, Oak Ridge National LaboratoryOak Ridge, TN, USA; ^2^Department of Ecology and Evolutionary Biology, University of TennesseeKnoxville, TN, USA; ^3^Bredesen Center for Interdisciplinary Research and Graduate Education, University of TennesseeKnoxville, TN, USA

**Keywords:** *Burkholderia*, *Pseudomonas*, *Populus deltoides*, microbiome, plant-microbe interactions

## Abstract

The biological function of the plant-microbiome system is the result of contributions from the host plant and microbiome members. The *Populus* root microbiome is a diverse community that has high abundance of β- and γ-*Proteobacteria*, both classes which include multiple plant-growth promoting representatives. To understand the contribution of individual microbiome members in a community, we studied the function of a simplified community consisting of *Pseudomonas* and *Burkholderia* bacterial strains isolated from *Populus* hosts and inoculated on axenic *Populus* cutting in controlled laboratory conditions. Both strains increased lateral root formation and root hair production in *Arabidopsis* plate assays and are predicted to encode for different functions related to growth and plant growth promotion in *Populus* hosts. Inoculation individually, with either bacterial isolate, increased root growth relative to uninoculated controls, and while root area was increased in mixed inoculation, the interaction term was insignificant indicating additive effects of root phenotype. Complementary data including photosynthetic efficiency, whole-transcriptome gene expression and GC-MS metabolite expression data in individual and mixed inoculated treatments indicate that the effects of these bacterial strains are unique and additive. These results suggest that the function of a microbiome community may be predicted from the additive functions of the individual members.

## Introduction

Plants are associated with a diverse microbiota composed of thousands of interacting organisms that must be considered when studying plant function (Vandenkoornhuyse et al., 2015). The plant microbiome can modify nutrient acquisition (Jeong and Guerinot, 2009; Behie et al., 2012; Rousk et al., 2013; Oteino et al., 2015), pathogen resistance (Berendsen et al., 2012; Weston et al., 2012) and host ability to tolerate stress (Fernandez et al., 2012; Lau and Lennon, 2012). However, these functionalities are often realized through interactions with an exceedingly complex metagenome, consisting of millions of bacterial genes often with unknown host consequences (Sessitsch et al., 2011; Knief et al., 2012). While predicting how the microbiome may function via metagenome sequencing is an important and informative approach, significant technical challenges exist and complementary functional validation studies of bacterial community remain necessary. To understand microbiome function and its concomitant genetic systems, it is beneficial to simplify the system by selecting small numbers of sequenced bacterial isolates to build surrogate microbiomes for direct experimentation.

Individually, cultured bacterial strains have been shown to have beneficial effects on host plants. A classic example of plant-bacterial symbiosis occurs between plant roots and bacterial strains, in which root cells recognize bacterial cells and form structures which facilitate the ability of bacterial associates to fix atmospheric nitrogen, providing a source of nitrogen for both the bacteria and host plant (Desbrosses and Stougaard, 2011; Chanway et al., 2014; Knoth et al., 2014; Lira et al., 2015). Beyond nitrogen fixation, bacteria have also been shown to benefit plant growth by increasing phosphorous uptake, ultimately impacting overall plant growth and biomass allocation (Vyas and Gulati, 2009; Oteino et al., 2015). Bacterial strains have also been shown to modify plant root growth through hormone signaling (Ryu et al., 2003) and increase disease resistance by stimulating the immune response of the host (Berendsen et al., 2012; Weston et al., 2012). Numerous other reports (Hardoim et al., 2008; Bulgarelli et al., 2013; Coleman-Derr and Tringe, 2014; Guttman et al., 2014; Laksmanan et al., 2014; Lebeis, 2014) have outlined the diverse set of mechanisms through which individual bacteria can promote the growth of their host plants.

As a community, the microbiome can have complex and synergistic effects on the host. For example, it has been shown that the microbiome can adapt to drought more rapidly than the host plant, and thus increase drought tolerance in *Brassica napus* (Lau and Lennon, 2012). The microbial community associated with the plant is dependent upon microenvironment (Lundberg et al., 2012; Ottesen et al., 2013; Edwards et al., 2015), soil properties and chemical environment (DeAngelis et al., 2009), soil microbiome (Zarraonaindia et al., 2015), plant host species (Weinert et al., 2011), and plant host physiological status (Zheng et al., 2015). However, the study of the natural microbiome poses several challenges, including the inability to maintain or culture many members of the natural microbiome. Recent work using a phylogenetically broad combination of cultured bacterial isolates showed differential leaf and root microbiota, indicating that community composition is dependent on genomic functional content of the microbial community members rather than phylogenetic lineage (Bai et al., 2015).

To determine how beneficial effects are combined in the plant microbiome, we selected two bacterial strains isolated from *Populus deltoides* hosts and studied how these bacteria benefit the host alone or in combination. The bacterial strains modified plant root architecture in both *Arabidopsis* and *Populus*. In *Populus*, we observe an increase in photosynthetic activity compared to uninoculated controls. Further, gene expression profiles and metabolite profiles changed differentially in individual and combination culture. This example supports the hypothesis that it is possible to predict the microbiome community phenotype based on experimentally determined phenotypes of individual microbial members.

## Methods

### Bacterial and plant culture

*Pseudomonas* GM41 and *Burkholderia* sp. BT03 bacterial strains were isolated using 3 rounds of colony-restreaking on rich medium (R2A, Franklin Lakes, BD Difco, NJ, USA) agar plates from surface-sterilized *Populus deltoides* roots from plants growing near the Caney Fork river in central Tennessee, USA (Gottel et al., 2011). Genomes were sequenced and assembled at Oak Ridge National Laboratory (Brown et al., 2012; Utturkar et al., 2014) and are available at NCBI (GM41: AKJN00000000.2; BT03: NZ_AKKD00000000.2) and IMG (GM41 genome ID: 2511231013; BT03 genome ID: 2562617112). Strains were maintained using R2A liquid or agar medium. To prepare for inoculation, strains were grown overnight in R2A medium at 25°C and 200 rpm shaking. Bacterial suspensions were washed 2X with 10 mM MgSO_4_ then diluted to OD_600_ = 0.01 for inoculation; 10 mL of inoculum was added to plant soil immediately prior to planting.

*Arabidopsis thaliana* Col-0 seeds were surface sterilized, plated on agar plates composed of 1/2X MS salts (Caisson Laboratories, Logan, UT, USA), 0.7% phytagar (Caisson Laboratories, Logan, UT, USA) 0.1% sucrose, stratified at 4°C for 2 d, and then moved to a growth chamber for germination. After 4 d, seedlings of equal size were transferred to fresh agar plates of the same media composition and bacterial strains were streaked 1 cm below roots. Plates were incubated for 7 d and then plants were imaged using Zeiss Axiovert SteREO microscope (Carl Zeiss AG, Pleasanton, CA, USA) to measure main and lateral root lengths and count root hairs. This assay was repeated in biological duplicate, using 3–4 plants per plate (total *n* = 12 plants each).

*Populus deltoides* (genotype “WV94,” ArborGen Inc. Ridgeville, SC, USA or genotype “B819”) shoot tips were sterilized by washing 5 min in 1% Tween 20, 1 min in 70% EtOH, 12 min in 0.6% NaOCl, then rinsed for 5 min 3X in sterile DI water. Cuttings were transferred to tissue culture medium containing 1X strength MS salts (Caisson Laboratories, Logan, UT, USA), 0.5% activated charcoal (Sigma-Aldrich, St. Louis, MO, USA), 2% sucrose, 0.05% MES (Sigma-Aldrich, St. Louis, MO, USA), 0.15% Gelrite (Plant Media, Dublin, OH, USA) and 0.1% PPM (Plant Cell Technology, Washington, DC, USA) and used as stock plants for up to 3 rounds of sub-culture. Sub-cultured plants were grown in the same tissue culture medium described above for 3 weeks until rooted, then transplanted into experimental condition media.

### Plant inoculation and harvest

Tissue culture rooted cuttings were planted in sterilized 3 × 3 × 4” polycarbonate vessels with couplers to double headspace and containing 120 cm^3^ inert clay (Pro's Choice Rapid Dry, Alpharetta, GA) and 80 mL 1X Modified Hoagland's nutrient solution (Phytotechnology Laboratories, Overland Park, KS, USA) that were subsequently inoculated with 10 mL of bacterial inoculation suspension. Plants were grown for 21 days in growth chambers under 16 h light, 8 h dark per day with ~50% humidity. Plants were harvested by carefully removing the entire plant from the soil, rinsing the root system in sterile DI water to remove loose soil, imaged and tissue was processed. Apical stem and leaves were flash-frozen immediately in liquid nitrogen and stored at −80°C. Root material was flash-frozen immediately in liquid nitrogen and stored at −80°C. Experiments were repeated twice with 5 plants per treatment per experiment. Phenotype data was analyzed using a two-way ANOVA with microbial inoculation as factors.

### Plant physiological measures

Prior to removal from soil, plant vessels were opened and leaves were dark-adapted for 25 min; physiological measures were then collected with a FluorPen 100 instrument (Photon Systems Instruments, Drasov, Czech Republic) according to manufacturer's instructions. Plants were then removed from soil matrix, washed briefly in sterile water to remove soil and then roots were spread for imaging. Root area was measured by thresholding image then counting white pixels using ImageJ (Schneider et al., 2012). Plant tissues were frozen in liquid nitrogen and stored at −80°C for molecular analysis.

### Nucleic acid extraction and qPCR

DNA was extracted from roots using PowerPlant kit (MOBIO, Carlsbad, CA, USA); tissue lysis and homogenization was obtained using three rounds of alternating bead beating (1 min) and liquid nitrogen freezing, followed by extraction according to manufacturer's instructions. For microbial quantification, qPCR primers were designed to detect *Pseudomonas* GM41 (forward 5′-ATCCGTACCATTTATGTTGATGAGT-3′ and reverse 5′-GAAACACATCCTCTTCGTTCTGTAT-3′) and *Burkholderia* BT03 (forward 5′-AGACTTCTTTGATTGAGGTGAAGTA-3′ and reverse 5′-CATATAGTCGAGATGGTCATTTAGG-3′) in mixtures. qPCR for bacterial quantification was performed using the iTAQ kit (Biorad, Hercules, CA, USA) on a CFX96 system according to manufacturer's instructions using bacterial genomic DNA as standard for quantification.

Stored leaf tissue was ground in liquid nitrogen and total RNA was extracted using a combined CTAB lysis buffer method and a Spectrum Total Plant RNA extraction kit (Sigma-Aldrich, St. Louis, MO, USA). Approximately 100 mg of flash-frozen ground tissue was incubated in 850 μL of CTAB buffer (1.0% β-mercaptoethanol) at 56°C for 5 min, 600 μL chloroform:isoamylalcohol (24:1) was added and samples were spun at 14,000 g for 8 min. The supernatant was removed and applied to the Spectrum kit filter column. RNA was precipitated in 500 μL of 100% EtOH and applied to the Spectrum kit binding column, and subsequent washes and elution were completed according to manufacturer instructions, including the optional on-column DNase treatment to rid the samples of residual genomic DNA. RNA quality and quantity were determined using a Nanodrop 1000 Spectrophotometer (Thermo Scientific, Waltham, MA, USA). A RevertAid first strand cDNA synthesis kit (Thermo Scientific, Waltham, MA, USA) was used to synthesize cDNA from 3 μg of total RNA for subsequent qPCR analysis (primer sequences available in Supplemental File S3). qPCR reactions for plant targets were done using SYBR Green with ROX (Biorad, Hercules, CA, USA) according to the manufacturer's instructions and reactions were run on an Applied Biosystems 7900HT instrument (Applied Biosystems, Foster City, CA, USA).

### RNA sequencing and analysis

Total RNA (1 μg) was sent to Macrogen (Seoul, South Korea) for *n* = 3 biological replicates, where libraries were prepared and sequenced on an Illumina HiSeq2000. All raw data was deposited at SRA database under accession number SRX1569823. The data handling and processing was performed based on our pipeline (Nookaew et al., 2012). The raw reads were first evaluated for quality using SolexaQA++ tool kits (Cox et al., 2010). The high quality reads (phred score > 25 and length after trimming > 25) were obtained using BWA dynamic trimming algorithm in the SolexaQA++ tool kits, aligned to the *Populus trichocarpa* v3.0 genome using bowtie2 (Langmead and Salzberg, 2012) and then used to generate read counts for statistical analysis. The WGCNA analysis workflow was used to cluster samples (Langfelder and Horvath, 2008). Differentially regulated genes were identified using the false discovery rate method with α = 0.2 for genes represented in all samples. MapMan software was used for analysis and statistical testing for pathway differential expression (*p* < 0.05, Wilcoxon Rank Sum test, Benjamini-Hochberg correction). The top 100 genes for each condition (sorted by *p*-value) were chosen for manual pathway annotation against the TAIR database.

### Metabolomics

Stored leaf tissue was ground in liquid nitrogen and twice extracted overnight with 2.5 mL of 80% ethanol in water at room temperature. Sorbitol was added (to achieve 10 ng/μL final concentration) before extraction as an internal standard to correct for differences in extraction efficiency, subsequent differences in derivatization efficiency and changes in sample volume during heating. Extracts were pooled and 1 mL of the extract was dried using a nitrogen stream. Dried extracts were dissolved in acetonitrile followed by the addition of N-methyl-N-trimethylsilyltrifluoroacetamide (MSTFA) with 1% trimethylchlorosilane (TMCS) and samples were then heated for 1 h at 70°C to generate trimethylsilyl (TMS) derivatives (Li et al., 2012; Tschaplinski et al., 2012). After 2 days, aliquots were injected into an Agilent 5975C inert XL gas chromatograph-mass spectrometer (GC-MS). The standard quadrupole GC-MS is operated in the electron impact (70 eV) ionization mode, targeting 2.5 full-spectrum (50-650 Da) scans per second, as described previously (Tschaplinski et al., 2012). Metabolite peaks were extracted using a key selected ion, characteristic m/z fragment, rather than the total ion chromatogram, to minimize integrating co-eluting metabolites. The extracted peaks of known metabolites are scaled to the total ion current using predetermined scaling factors. Peaks were quantified by area integration and the concentrations normalized to the quantity of the internal standard recovered, amount of sample extracted, derivatized and injected. A large user-created database (~2300 spectra) of mass spectral electron impact ionization (EI) fragmentation patterns of TMS-derivatized compounds, as well as the Wiley Registry 10th Edition combined with NIST 2014 mass spectral library, were used to identify the metabolites of interest to be quantified. There were 3 replicate plants per treatment and treatment differences were tested for statistical significance (*p* ≤ 0.05) using Student's *t*-tests. Data are presented as log2(fold change), which is calculated by determining fold change defined as absolute value of change up or down, then scaled by taking the logarithm of the data and applying positive or negative to indicate increase or decrease in expression, respectively. The GCMS data generated in this project has been uploaded to the MetaboLights database (ebi.ac.uk/metabolights) with accession number MTBLS332.

## Results

### Functional characterization of two bacterial endophyte isolates from *P. deltoides*

We chose two bacterial strains based on diverse taxonomy and abundant representation in the *Populus* microbiome (Gottel et al., 2011; Shakya et al., 2013) to study individual and cooperative effects on host plant growth (Table 1). Both endophytic bacterial strains were isolated from surface-sterilized *Populus* fine-roots collected from the same field site in central Tennessee, USA (Gottel et al., 2011). Genome sequencing and quality for these strains was described previously (Brown et al., 2012; Utturkar et al., 2014). *Burkholderia* BT03 is most closely related to *Burkholderia terrae* BS001 (IMG average nucleotide identity clique analysis) and *Pseudomonas* GM41 is a member of the *Pseudomonas fluorescens* clade (Timm et al., 2015; Jun et al., 2016). Based on genome annotation evidence both strains encode functions potentially important in plant growth promotion and colonization including ACC deaminase, phytohormone production, pili, flagella, chemotaxis machinery, signal transduction, and secretion systems. The genome of BT03 shows higher occurrence of transposase elements, likely contributing to the larger genome size and gene count through horizontal gene transfer. Additional functional elements include synthesis, degradation and transport of secondary metabolites, the majority of which are predicted to be involved in the degradation of aromatics, metabolites common in the poplar metabolome and prevalent in other plant-associated *Burkholderia* (Suárez-Moreno et al., 2012). There are also additional genes in BT03 for carbohydrate degradation and lipid transport and metabolism, indicating the potential to modulate cell surface presentation, a strategy used by some pathogens to avoid the host immune response. Specifically, a wcaH homolog (GDP-mannose mannosyl hydrolase) is predicted in the BT03 genome and encodes the enzyme for synthesis of colanic acid, which has been shown to be important for attachment to cell membranes (Hanna et al., 2003) and has also been observed in bacterial strains associated with wood products (Rättö et al., 2006). The metabolic capabilities of *Pseudomonas* GM41 have been described elsewhere (Timm et al., 2015), but notable functions include sucrose and 2,3-dihydroxybenzoate degradation, siderophore production, the ability to solubilize phosphate and use nitrate as an electron acceptor (denitrification) and production of the plant hormone indole-3-acetic acid. The GM41 genome has also increased abundance of cell motility genes, nucleotide metabolism and replication, potentially indicating a faster growth lifestyle compared to BT03. A full list of COG categories by genome is available in Supplemental File S1.

**Table 1:** **Bacterial strains used in this study Genome statistics extracted from IMG (img.jgi.doe.gov)**.

**Strain**	**BT03**	**GM41**
Class	β-*Proteobacteria*	γ-*Proteobacteria*
Genus	*Burkholderia*	*Pseudomonas*
genome size (Mb)	10.9	6.6
gene count (COG)	7779	5082
**COG Functional Category**	**Count**	
Mobilome: prophages, transposons	124	43
Secondary metabolites biosynthesis, transport, and catabolism	337	148
Carbohydrate transport and metabolism	582	291
Lipid transport and metabolism	489	250
Energy production and conversion	644	349
Transcription	853	475
Cell wall/membrane/envelope biogenesis	478	270
General function prediction only	794	504
Inorganic ion transport and metabolism	390	263
Coenzyme transport and metabolism	359	250
Intracellular trafficking, secretion, and vesicular transport	130	93
Defense mechanisms	158	118
Amino acid transport and metabolism	757	571
Signal transduction mechanisms	379	297
Replication, recombination and repair	147	121
Function unknown	334	283
Posttranslational modification, protein turnover, chaperones	223	197
Cell motility	136	123
Nucleotide transport and metabolism	116	109
Translation, ribosomal structure and biogenesis	260	246

*Highlighting indicates functions that are present in higher fractional abundance in genome*.

**Figure 1:**
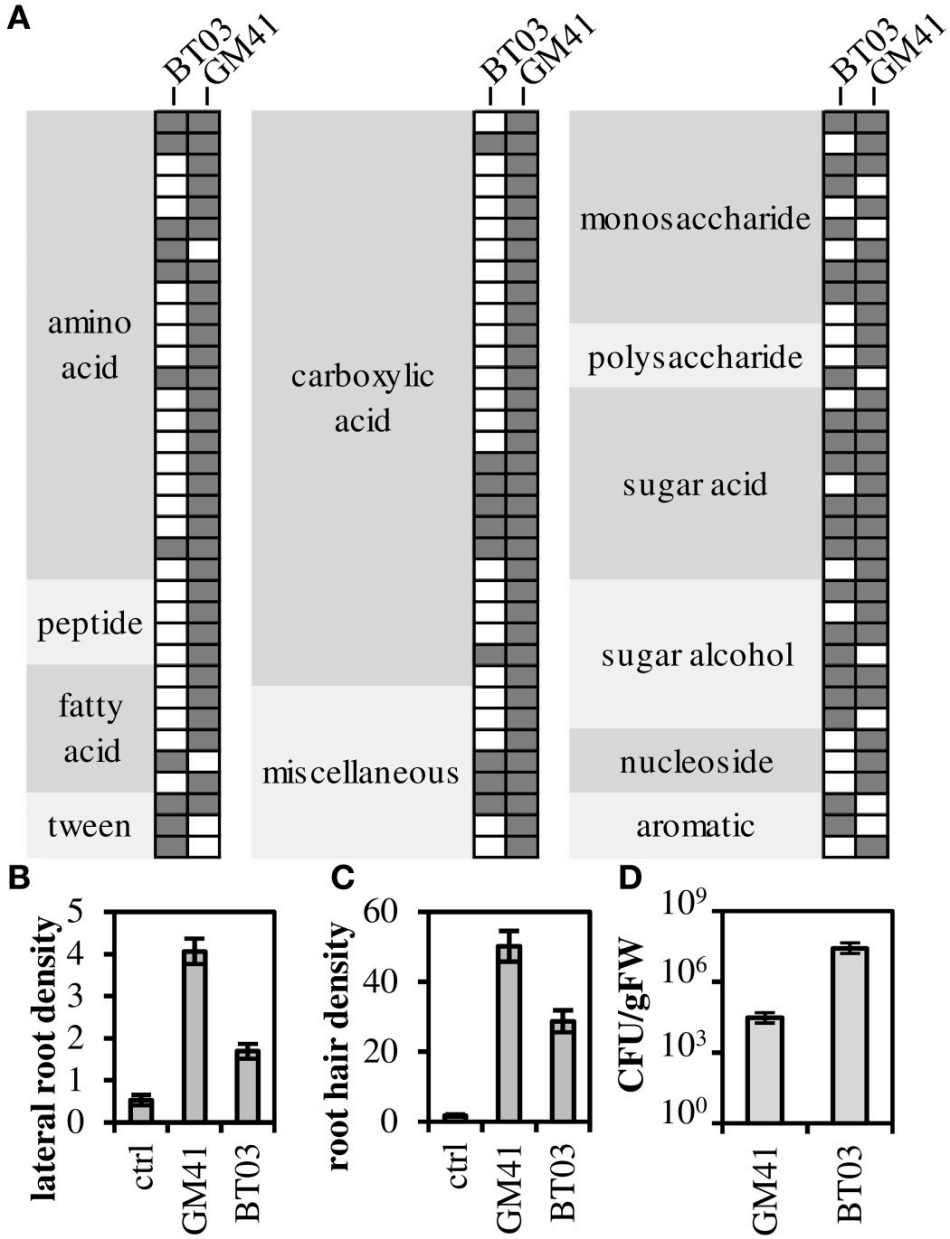
**Functional characterization of strains**. **(A)** Filled spot indicates growth as measured by absorbance at 600 nm (OD_600_). **(B,C)** Strains were grown with *A. thaliana* in plate assays to determine root phenotype: lateral root density (number per cm main root) and root hair density (number per cm root) Error bars are SEM from *n* = 12 plants. All values are significantly different at *p* < 0.001 (Student's *t*-test). **(D)** *Populus* (genotype “B819”) root colonization density. Error bars are SEM from *n* = 5 plants. Colonization density is significantly different at *p* < 0.01.

Both strains were metabolically characterized by the ability to grow on a panel of sole carbon sources (Figure 1A, Supplemental File S2). Though *Burkholderia* BT03 grew on fewer amino acid and carboxylic acid substrates, it showed similar or enhanced ability to grow on sugars and sugar derivatives, consistent with identified genomic pathways noted above (and listed in Supplemental File S1). BT03 has additional pathways for degradation of chitin, cellulose and pectin, indicating potential to grow on complex carbon sources, while GM41 grows on more small metabolites available in the endosphere environment, suggesting that BT03 and GM41 may occupy a different metabolic niche in the host plant. *Burkholderia* BT03 and *Pseudomonas* GM41 both increased lateral root density and hair density in *Arabidopsis* plate assays but to different quantitative extents (Figures 1B,C). BT03 also heavily colonized roots in this assay, while GM41 tended to grow visually observable colonies on a limited section of the roots (Figure S1). These results indicate different mechanisms and/or niche specialization leading to complex phenotypes of increased lateral root and root hair production. Finally, these strains individually colonized axenic *Populus* plants to different levels, with BT03 colonizing at 3 × 10^7^ CFU/g root and GM41 colonizing roots at 3 × 10^4^ CFU/g root (Figure 1D). This enhanced endophytic colonization ability of BT03 could be due to its additional genomic encoded secretion systems, host cell wall hydrolases, outer membrane polysaccharides, inorganic ion transport systems and carbon substrate utilization pathways (Table 1).

### Endophytes increase root area and photosynthetic capability

**Figure 2:**
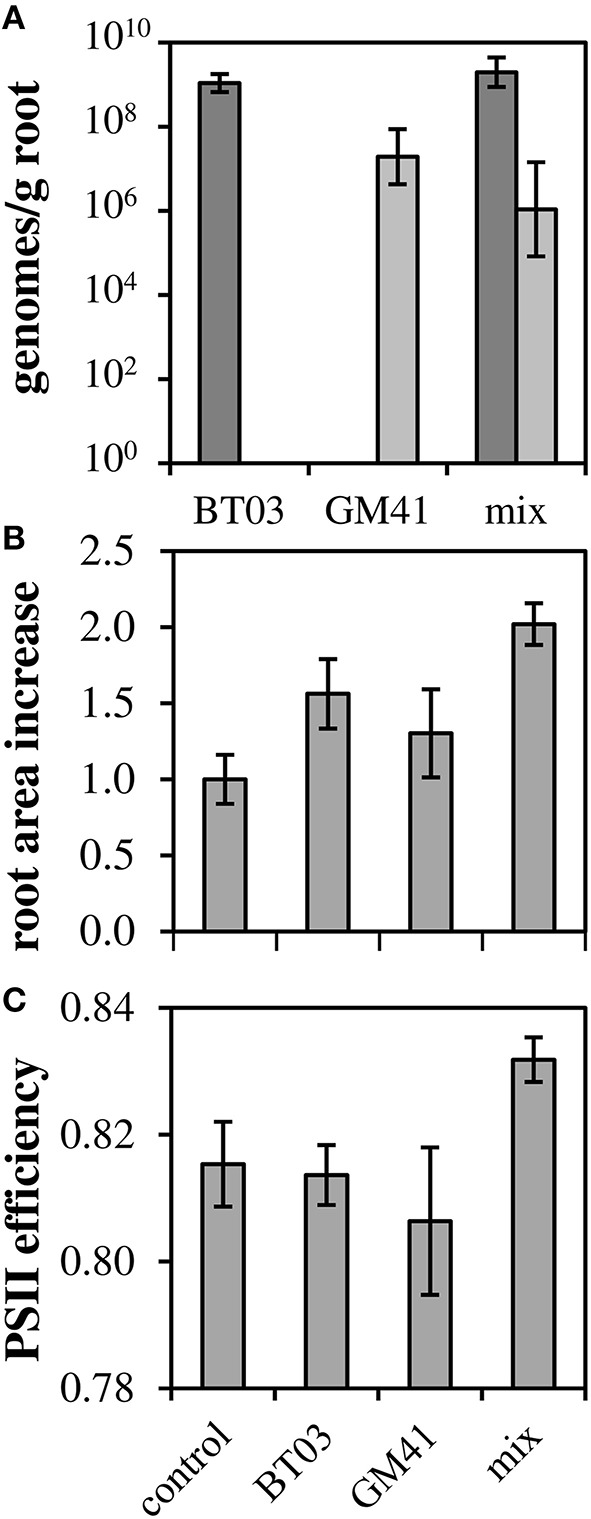
**Co-colonization density and increases in root biomass and photosynthetic ability**. **(A)** Root colonization density of bacteria measured using single-copy gene qPCR. Light bars, GM41; Dark bars, BT03. Error bars are 1 SD from *n* = 3 plants. **(B)** Root area increased normalized to control (ΔArea_Test_/ΔArea_Control_). **(C)** QYmax, a measure of photosynthetic efficiency defined as maximum quantum yield of PSII in dark-adapted state. For **(B,C)**, Error bars are standard error for *n* = 10 plants, 2-way ANOVA was used for statistical test and is discussed in text.

To determine how these strains affect the host plant individually and in a community, we measured phenotypes for uninoculated controls, individually inoculated plants and dual-inoculated plants (*n* = 2 biological replicates, 5 plants per treatment). Axenic, rooted cuttings of *Populus deltoides* were inoculated with *Burkholderia* BT03, *Pseudomonas* GM41, or both under sterile conditions. Plants were grown for 21 days in sealed containers in growth chambers and then assayed for root colonization and host phenotypes including photosynthetic potential and host gene expression. *Burkholderia* sp. BT03 colonized the roots at ~10^9^ genomes/g FW root, while GM41 colonized at 10^7^ genomes/g FW root using strain-specific single-copy gene qPCR assay to distinguish strains. Colonization rates were similar in mono- or dual-inoculated plants, suggesting little to no competitive interaction between bacteria as measured by abundance (Figure 2A).

Both strains significantly increased root growth in the host relative to control, with ANOVA results indicating no bacteria-by-bacteria interaction (Figure 2B), indicating that the increase in root growth in dual-inoculated plants can be attributed to the additive effects of these strains. Photosynthetic potential, as measured by the maximum quantum yield of photosystem II, increased to 83% relative to uninoculated controls and was significant in dual-inoculated plants only (Figure 2C, *p* < 0.05). We attribute this increase to the increased root area observed in dual inoculated plants.

### Transcriptome analysis indicates unique host effect and complementary effect in mixture

**Figure 3:**
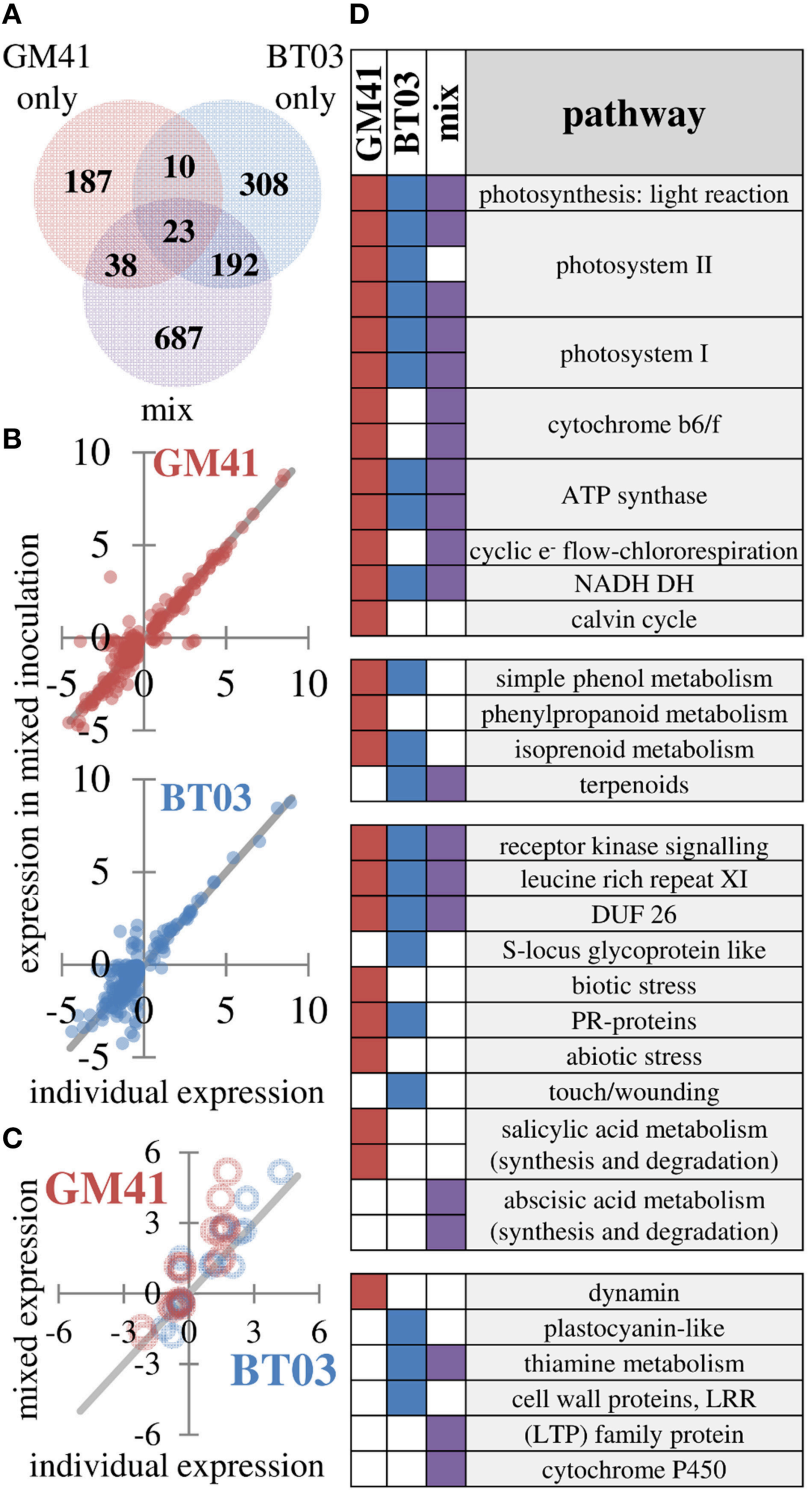
**Changes in gene expression indicate different responses to strains which are detected in mixture**. **(A)** Summary of 1445 genes identified as differentially expressed using FDR = 0.2 **(B)** Individual expression levels (x-axis) vs. expression levels in mixture (y-axis) for differentially expressed genes in individual treatments. Blue points are *Burkholderia*, red points are *Pseudomonas*. Line indicates perfect correlation for comparison. **(C)** Expression correlation for 19 genes selected to confirm transcriptome patterns Blue points are *Burkholderia*, red points are Pseudomonas. Line indicates perfect correlation for comparison. **(D)** Differentially regulated pathways in response to microbial inoculation. Pathway level has been reduced for presentation (see Supplemental File S3 for complete dataset) A filled square indicates significant change in indicated pathway (*p* < 0.05, Wilcoxon rank sum test, Benjamini Hochberg correction).

Plant gene expression response was measured in apical leaf tissue using whole-transcriptome sequencing analysis, based on observed changes in photosynthesis and limitations in extraction of root RNA and metabolites. Reads were aligned to the *Populus trichocarpa* v3.0 genome, the closest genome-sequenced relative of *Populus deltoides* plants used in this study (81–86% of reads aligned). Additional alignment statistics are included in Supplemental File S3. Between sample expression data generally clustered into two groups, i.e., uninoculated control and bacterial treatments (Figure S2). Of the 26,168 genes represented in all samples, 1445 were identified as differentially expressed (FDR = 0.2, *p* < 0.01) in at least one of the bacterial treatments (*Pseudomonas, Burkholderia* or mixture; Figure 3A, complete list in Supplemental File S3). As shown, 23 genes were up- or down- regulated in all treatments. We observe 38 genes that were shared between *Pseudomonas* and the mixture and 192 genes shared between *Burkholderia* and the mixture, with only 10 shared between BT03 and GM41, suggesting strain-specific transcriptional response of the host plant to individual strains independent of mono- or dual- inoculation. To further test the hypothesis that strains activate unique genes individually and in the mixture, we plotted expression of all genes differentially regulated in *Pseudomonas* or *Burkholderia* treatments versus expression in the mixed inoculation (Figure 3B). We observe agreement between individual and mixed conditions. Genes were sorted by ascending *p*-value, and the top 100 most significant genes for each treatment were then classified into functional pathways by manual curation with TAIR, using data only from mechanistic and phenotypic reports. We observed changes in abscisic acid, light response, oxidative stress and pathogen defense in response to all treatments (Supplemental File S3). Light response and chlorophyll production is consistent with the observed increase in photosynthetic ability. *Burkholderia* induced changes in auxin and benzoate production, as well as root growth and root hair development genes. *Pseudomonas* GM41 induced changes in cell wall and cold stress response. These functional changes were also observed at similar rates in the mixed culture condition. We did observe changes in response to individual microbes not seen in the mixture, including ethylene response, heat stress and salt stress response. Equally, we observe functions that were induced in the presence of both bacteria only, including lipid, sulfate, and thiamin biosynthesis.

Genes detected in all samples were used for pathway analysis using MapMan (Usadel et al., 2009). Consistent with increased photosynthesis, the light reaction pathway was significantly changed by GM41, BT03, or mixed inoculation (*p* < 0.05). While GM41 affected all pathways shown for the light reaction, BT03 did not modify expression of cytochrome b6/f, chlororespiration or the Calvin cycle. The majority of pathways involved in the light reaction were also regulated in the mixed inoculation. Inoculation with BT03 affected cell wall proteins, thiamine metabolism, the touch/wounding pathway within abiotic stress, plastocyanin-like pathway involved in photosynthesis and lipid metabolism (Figure 3D, Supplemental File S3). GM41 inoculation uniquely modified phenylpropanoid metabolism, the salicylate pathway, both biotic and abiotic stress and dynamin metabolism (*p* < 0.05, Figure 3D). Transcriptome data was supported by qRT-PCR for 19 genes in the calmodulin, stress and detoxification pathways, cell wall modification, pathogen defense, ethylene, jasmonic acid, and salicylate pathways and secondary metabolism (Figure 3C and Figure S3). Notably, we observed overall decrease in expression of defense related protein in BT03 inoculation, consistent with the hypothesis that BT03 modulates host defense.

### Metabolite profile in dual-inoculated plants correlates with single inoculation results

**Figure 4:**
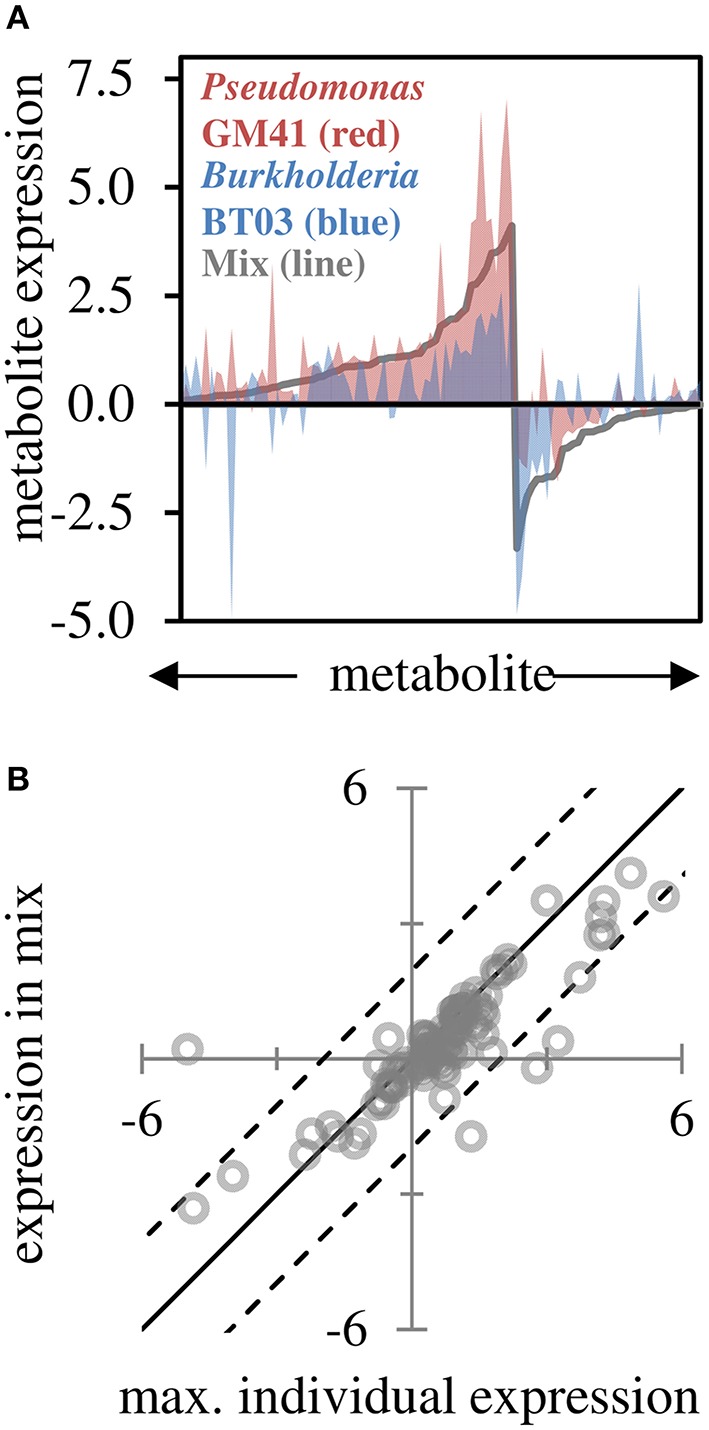
**Leaf metabolite changes for GM41, BT03, or dual inoculated plants. (A)** Log2 fold-change calculated from fold-change up or down for treatment relative to control (*n* = 3 each) for 103 metabolites, sorted by fold change in mixed inoculation. Red indicates *Pseudomonas* inoculated plants, blue indicates *Burkholderia* inoculated plants, and line indicates mixture. **(B)** Maximum individual expression for each metabolite plotted vs. expression in mixture. Line indicates perfect correlation for comparison, dashed lines are 95% confidence intervals of the data.

Based on GC-MS characterization of leaf metabolites, we quantified the relative area of 103 unique metabolite peaks (Figure 4A, Supplemental File S4). The metabolic profile of the dual-inoculated plants tended to reflect the most extreme change induced by either of the individual strains (Figures 4A,B). Consistent with qPCR and RNAseq, there were increases in lipid precursors, palmitic acid, linoleic acid, and azeleic acid in inoculated plants, indicative of lipid peroxidation (Zoeller et al., 2012). Amino acids and sugar (glucose, fructose and sucrose) concentrations in leaves were decreased, potentially indicating transport of photosynthate from the leaf tissue in support of the observed increased root growth. In the dual-inoculated plants we uniquely detected decreases in metabolic precursors of secondary metabolite pathways including shikimate, quinate, ferulate, tryptophan and multiple unidentified glycosides and aromatics. In BT03 inoculated plants, based on RNAseq and RT-qPCR, the JA pathway was upregulated and we documented increases in the jasmonic acid precursor α-linolenic acid in both the BT03 and mixed inoculum plants. Salicylate concentration was increased in response to all microbial inoculations, with the highest increased in GM41 inoculated plants. Catechin was up-regulated 1.3X in the mixed inoculations and -1.7X in BT03 inoculation. B-cyano-alanine and shikimate were also increased significantly in single-inoculation treatments, but the mixture showed only modest changes in these metabolites.

## Discussion

The function of a plant-microbiome system is the result of complex interactions starting with cell-to-cell communications that can cascade through multiple levels of biological organization including tissue, organ and systemic whole-plant responses. The prevalence of *Proteobacteria* in the microbiomes of diverse plants (Bulgarelli et al., 2013), and specifically the high abundance of γ- and β- *Proteobacteria* in the *Populus* microbiome (Gottel et al., 2011; Shakya et al., 2013) motivated us to choose two representatives from our isolate collection to study effects in individual and mixed culture. To understand the function of communities of organisms and their resulting interactions, and based on our results and others (Bodenhausen et al., 2014), we can generate simplified models of the microbiome using cultured representatives of the microbiome inoculated onto gnotobiotic plants under controlled laboratory conditions. The data presented here supports the hypothesis that the bacterial strains in this study occupy different niches and have unique mechanisms to interact with the host that are independent of the alternate strain, indicating that the community phenotype in this 3-member system is predictable based on contributions of individual bacterial strains.

The similar strain-specific abundances observed in mono- and dual-inoculation in this study indicate that the two bacterial strains used here occupy non-competitive niches in the host environment. While both strains were isolated from the endosphere, there is substantial structural and chemical variability within host roots that could provide unique functional roles for these bacterial strains (McCully, 2001). It is also likely that the lack of a complex rhizosphere microbiome allowed either or both of these strains to exist in the rhizosphere environment. The individual metabolic ability of each bacterial strain may facilitate colonization and growth on unique host metabolites. We did observe differences in metabolic versatility of these strains, such that, the *Pseudomonas* isolate able to grow on more carbon substrates than the *Burkholderia* isolate which is predicted from genome analysis to grow on polysaccharides and complex carbon sources. Further, the lack of additional competitors in the community may have fostered the observed niche differentiation. Investigating similar effects using microbes that may compete for niche space will allow us to determine how specific microbes colonize and compete for resources within the host, and how the plant maintains the beneficial effect from the microbe.

Overall, we observed activation of unique pathways by individual strains. *Burkholderia* species have been shown to produce stress hormones (Kurepin et al., 2015) and indole-3-acetic acid (Naveed et al., 2014), resulting in growth promotion of shoots and roots. Plant-associated *Burkholderia* strains encode multiple strategies for plant interaction (Mitter et al., 2013) and have also been shown to sense the plant in the environment and respond to stress (Sheibani-Tezerji et al., 2015). Specifically, *Burkholderia* BT03 increased root growth and stimulated expression of genes in the photosynthesis pathway, and phenol, terpenoid, and isoprenoid metabolism. BT03 also induced expression of pathogen response and cell wall modification. Transcriptome results were supported by metabolite data, for example leaf sucrose was decreased potentially indicating carbohydrate into roots, and linoleic, α-linoleic, and palmitic acids were increased in leaf tissue.While BT03 colonized at a high density relative to *Pseudomonas* GM41, we did not observe direct activation of the immune response by BT03, potentially due to evasion by the bacteria. The well-studied *Pseudomonas fluorescens* clade (Silby et al., 2009; Loper et al., 2012; Timm et al., 2015; Jun et al., 2016) encodes multiple mechanism for plant interaction, including iron acquisition, hormone production, plant sensing, and signaling through multiple secretion systems. *Pseudomonas* GM41 also increased root growth, and induced changes in photosynthesis, phenol and phenylpropadnoid metabolism. These changes were supported by increased in leaf amino acids and phenolic glycosides. GM41 also induced expression of the biotic and abiotic stress pathways and the salicylate pathway. Interestingly, many of the pathways induced by individual strains are also differentially regulated in the dual-inoculation. In *Arabidopsis* plate assays, both strains used in this study increased lateral root formation and root hair growth, though to different extents. In the native *Populus* host, both strains affected plant root growth, for which the interaction term was insignificant (two-way ANOVA) indicating that observed increases were the result of additive effects of the two strains. Photosynthetic pathways and kinase signaling were up-regulated by both strains individually and in the mixed inoculation. However, there were some cases where strains induced expression of pathways which were not observed in the mixture, such as pathogen response which has shown to be modulated by inoculation with beneficial *Psuedomonas fluorescens* strains (Weston et al., 2012). Overall, plant gene expression response to mixed inoculation appears to reflect the additive effect of the effects of the two individual strains. Metabolite data also showed additive response. However, for the metabolites detected the expression tended toward an intermediate expression level in response to the mixed inoculation. For example, glutmate was 4.9-fold increased in response to GM41 alone, and increased 3.2-fold in response to BT03, with the response to mixed inoculation at 4.1-fold. Together, the data suggests that the strains in this study provide unique and non-overlapping contributions to the host plant. Overall gene and metabolite expression indicates increased photosynthesis in response to microbial inoculation relative to un-inoculated controls. This data was supported by photosynthetic efficiency data, which was only increased significantly in the mixed inoculation. While these results are encouraging, in this case we present data using two diverse representative strain from a multi-member microbiome. Adding more bacterial strains with overlapping functionality may induce synergistic effects in the host plant. In more complex communities, competition from other bacteria could also increase interactions between strains and lead to more complex community phenotypes.

The results of this study provide a proof-of-concept example of how microbial-mediated beneficial host effects can be applied in an additive manner using low diversity constructed communities, providing an opportunity to predictively engineer the plant-microbiome relationship for targeted community functions. However, substantial research challenges remain. For example, the spatial, chemical and temporal resolution of the root and the root hair environment is not well characterized (McCully, 2001), nor do we have a good understanding of how host genetic factors like the host immune response (Lebeis, 2014; Lebeis et al., 2015) shapes endophyte colonization and abundance. In addition to direct effects of the host plant environment, spatial heterogeneity of the community resulting from dispersal or stochastic colonization, genetic diversity and functional overlap of community members, and cooperation and competition for resources with other microbiome members (Costello et al., 2012; Coyte et al., 2015) complicate predicting the phenotype of these communities. Methods developed in this study, such as effect of single microbial inoculation on gene-expression, metabolite production and host phenotype, together with future research on larger and more diverse constructed communities and host determinants of microbiome colonization will inform the design of these communities. Further work on constructed microbiome communities will help elucidate complex community phenotypes and will help realize the goal of engineering host phenotype through microbiome manipulation.

## Author contributions

Comparative bacterial genomics and phenotypes: CT, TL, MD, DP. Plant phenotypes: CT, SJ, LG, JH, JA, EG, GT, DW. Molecular biology and analysis: CT, SJ, IN, ZY, DW. Metabolomics and analysis: CT, NE, TT.

## Funding

This research was funded by the U.S. DOE Office of Biological and Environmental Research, Genomic Science Program under Plant Feedstock Genomics projects: DE-SC001043 (50%) and the Plant-Microbe Interfaces Scientific Focus Area (50%) at Oak Ridge National Laboratory.

### Conflict of interest statement

The authors declare that the research was conducted in the absence of any commercial or financial relationships that could be construed as a potential conflict of interest.
