# Supplementary material for: Two Poplar-Associated Bacterial Isolates Induce Additive Favorable Responses in a Constructed Plant-Microbiome System
Source: Front Plant Sci. 2016 Apr 26;7:497. doi: 10.3389/fpls.2016.00497 (PMC4845692; doi:10.3389/fpls.2016.00497)
Supplement: Supplementary file 1 [file Presentation1.PDF]

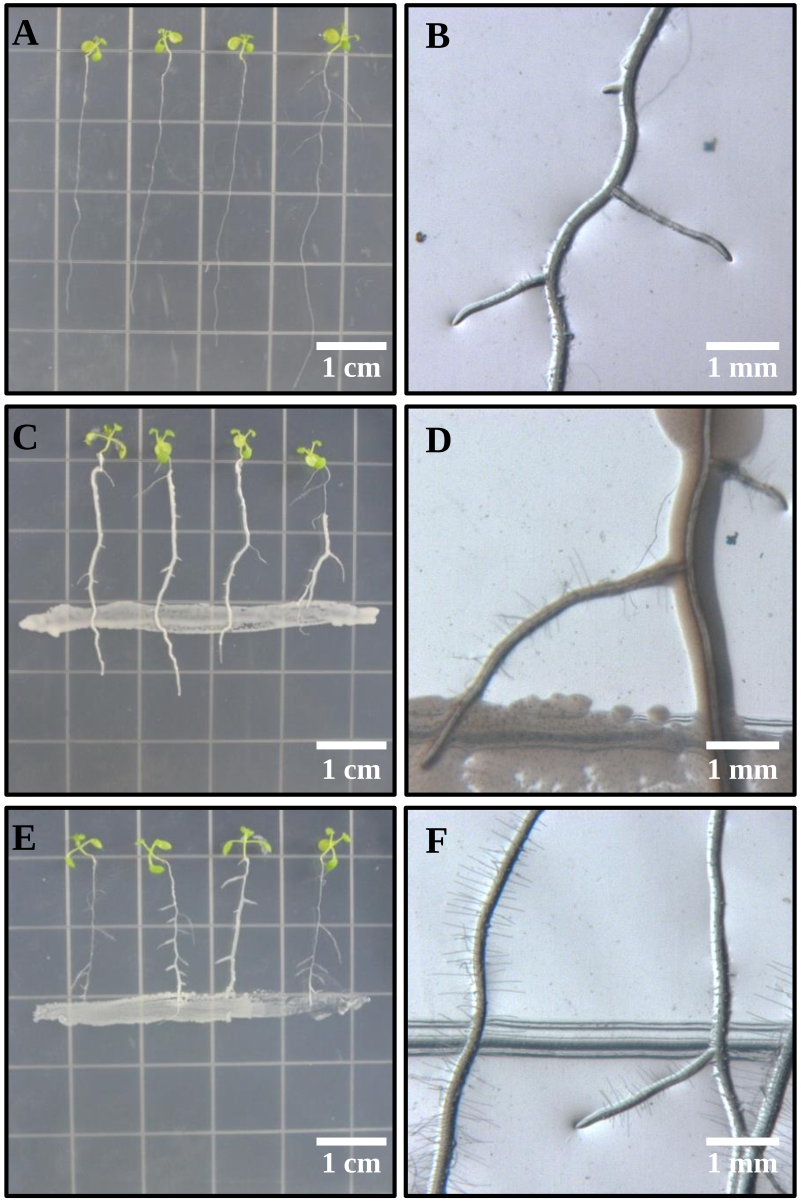

Figure S1: ***Arabidopsis*** **root phenotype plate assay**. Representative images of control (A-B), BT03 (C-D), and GM41 (E-F) root phenotypes.

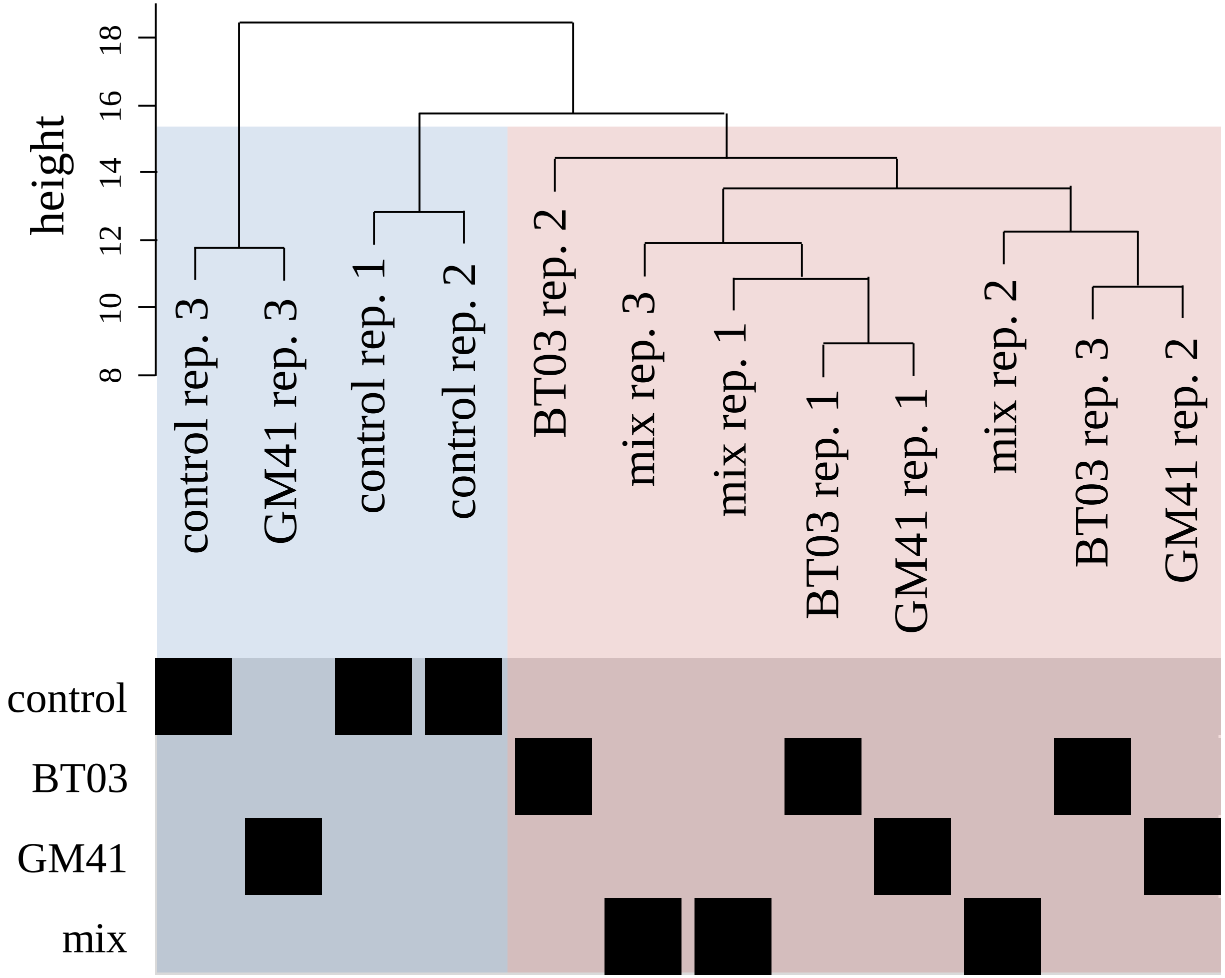

Figure S2: Sample clustering by expression Gene expression profiles were clustered using the WGCNA workflow. Blue and red shading indicates two groups. Black boxes show inclusion of treatments in groups.

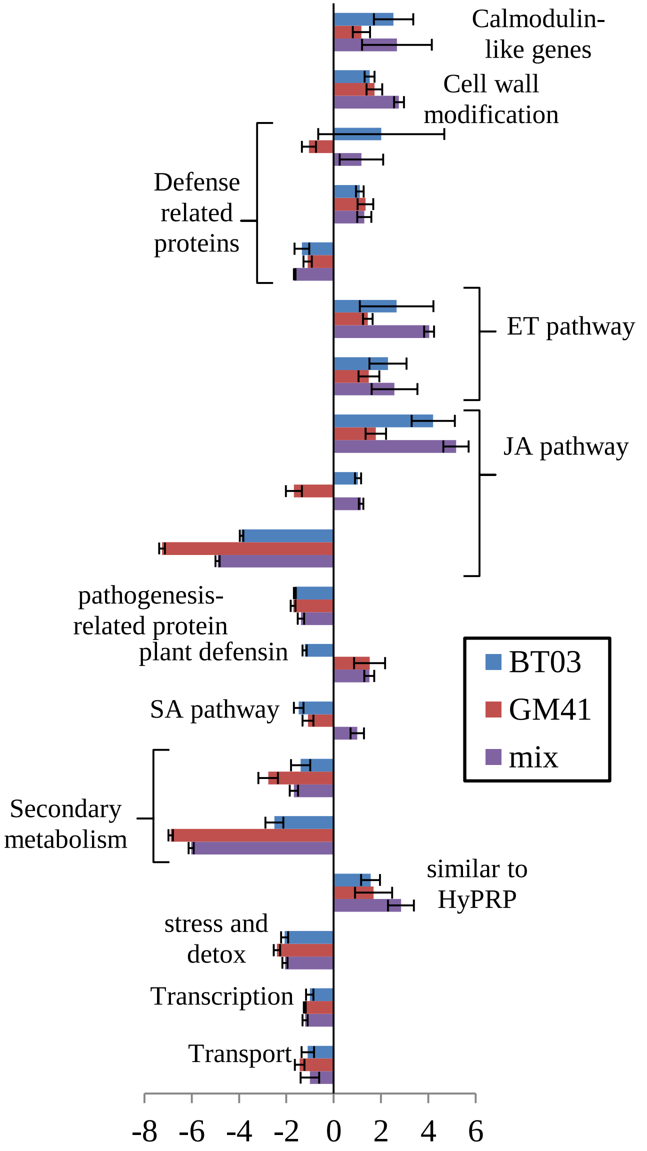

Figure S3: Gene expression panel shows similar response to inoculation. Effect of inoculation on plant gene expression. Error bars are standard error.
